# 旁系CMV阳性和CMV阴性供者对异基因造血干细胞移植结局的影响

**DOI:** 10.3760/cma.j.cn121090-20251016-00470

**Published:** 2026-08

**Authors:** 静 刘, 圆圆 张, 晓冬 莫, 于谦 孙, 萌 吕, 旭颖 裴, 晓甦 赵, 翼飞 程, 海霞 付, 兰平 许, 晓辉 张, 晓军 黄, 昱 王

**Affiliations:** 北京大学人民医院，北京大学血液病研究所，国家血液系统疾病临床医学研究中心，造血干细胞移植治疗血液病北京市重点实验室，北京 100044 Peking University People's Hospital, Peking University Institute of Hematology, National Clinical Research Center for Hematologic Disease, Beijing Key Laboratory of Hematopoietic Stem Cell Transplantation for Hematologic Diseases, Beijing 100044, China

**Keywords:** 造血干细胞移植, 供者选择, 巨细胞病毒, 预后, Hematopoietic stem cell transplantation, Donor selection, Cytomegalovirus, Prognosis

## Abstract

**目的:**

探索旁系巨细胞病毒（CMV）阳性和CMV阴性供者对异基因造血干细胞移植（allo-HSCT）结局的影响。

**方法:**

本项回顾性队列研究分析了2017年1月至2025年3月在北京大学人民医院接受CMV阴性单倍体供者（CMV阴性供者组）和旁系CMV阳性单倍体供者（旁系CMV阳性供者组）来源移植物的allo-HSCT患者的CMV相关事件和生存结局，患者的CMV血清学均为阳性。

**结果:**

本研究共纳入126例患者，来特莫韦在国内上市前，CMV阴性供者组16例，旁系CMV阳性供者组58例；来特莫韦在国内上市后，CMV阴性供者组27例，旁系CMV阳性供者组25例。中位随访1 091（660，1 536）d，来特莫韦在国内上市前，CMV阴性供者组难治性CMV血症发生率（87.5％对50.0％，*P*＝0.007）和首次CMV血症持续中位时间［29（95％*CI*：25～33）d对17（95％*CI*：15～19）d，*P*＝0.004］明显高于旁系CMV阳性供者组。来特莫韦的应用显著推迟了两组患者移植后首次CMV血症的发生时间：CMV阴性供者组从移植后37（95％*CI*：31～43）d延迟至140（95％*CI*：136～144）d（*P*<0.001）；旁系CMV阳性供者组从移植后36（95％*CI*：33～39）d延迟至移植后111（95％*CI*：76～146）d（*P*＝0.013）。应用来特莫韦的情况下，CMV阴性供者组和旁系CMV阳性供者组首次CMV血症持续中位时间的差异无统计学意义［18（95％*CI*：17～19）d和12（95％*CI*：0～25）d，*P*＝0.313］。来特莫韦的应用降低了CMV阴性供者组难治性CMV血症的发生率（40.7％对87.5％，*P*＝0.003），但仍高于旁系CMV阳性供者组（40.7％对12.0％，*P*＝0.020）。无论是否应用来特莫韦，CMV阴性供者组和旁系CMV阳性供者组3年总生存率、非复发相关死亡率、累积复发率的差异均无统计学意义（*P*值均>0.05）。多因素分析显示，CMV疾病是影响患者总生存（*HR*＝3.0，95％*CI*：1.3～7.3，*P*＝0.014）和非复发相关死亡（*HR*＝3.1，95％*CI*：1.1～8.8，*P*＝0.035）的独立危险因素。CMV疾病发生的独立危险因素为难治性CMV血症（*HR*＝12.0，95％*CI*：2.9～50.7，*P*＝0.001）。Logistic回归分析显示，难治性CMV血症发生的独立危险因素为无来特莫韦预防的CMV阴性供者（*P*＝0.005）。

**结论:**

CMV阴性供者会增加受者CMV相关事件的发生风险。来特莫韦的应用虽可改善这一情况，但CMV阴性供者组难治性CMV血症的发生率仍高于旁系CMV阳性供者组，两种供者来源的受者总体生存结局相当。

## Abstract (en)

**Objective:**

To investigate the impact of collateral cytomegalovirus（CMV）-positive versus CMV-negative donors on the outcomes of allogeneic hematopoietic stem cell transplantation（allo-HSCT）.

**Methods:**

This retrospective cohort study analyzed CMV-related events and survival outcomes in allo-HSCT recipients with CMV seropositive who received grafts from CMV-negative（CMV-negative donor group）or collateral CMV-positive（collateral CMV-positive donor group）haploidentical donors at Peking University People's Hospital from January 2017 to March 2025.

**Results:**

A total of 126 patients were enrolled, including 16 CMV-negative and 58 collateral CMV-positive donors before the availability of letermovir, and 27 CMV-negative and 25 collateral CMV-positive donors after the availability of letermovir. With a median follow-up of 1 091（660, 1 536）days, before the availability of letermovir in China, the CMV-negative donor group had a significantly higher incidence of refractory CMV viremia（87.5％ *vs* 50.0％, *P*＝0.007）and a longer median duration of first CMV viremia［29（95％ *CI*: 25–33）*vs* 17（95％ *CI*: 15–19）days, *P*＝0.004］than the collateral CMV-positive donor group. The use of letermovir significantly delayed the onset of first post-transplant CMV viremia in both groups: from day 37（95％ *CI*: 31–43）to day 140（95％*CI*: 136–144）in the CMV-negative donor group（*P*<0.001）and from day 36（95％ *CI*: 33–39）to day 111（95％ *CI*: 76–146）in the collateral CMV-positive donor group（*P*＝0.013）. With letermovir prophylaxis, the difference in the median duration of the first CMV viremia between the CMV-negative and collateral CMV-positive donor groups was no longer significant［18（95％*CI*: 17–19）*vs* 12（95％*CI*: 0–25）days, *P*＝0.313］. Letermovir reduced the incidence of refractory CMV viremia in the CMV-negative donor group（40.7％ *vs* 87.5％, *P*＝0.003）, but the incidence remained higher than that in the collateral CMV-positive donor group（40.7％ *vs* 12.0％, *P*＝0.020）. Regardless of letermovir use, there were no statistically significant differences in the 3-year overall survival, non-relapse mortality, or cumulative relapse rate between the CMV-negative and collateral CMV-positive donor groups（all *P*>0.05）. Multivariate analysis showed that CMV disease was an independent risk factor for overall survival（*HR*＝3.0, 95％*CI*: 1.3–7.3, *P*＝0.014）and non-relapse mortality（*HR*＝3.1, 95％*CI*: 1.1–8.8, *P*＝0.035）. Refractory CMV viremia was an independent risk factor for CMV disease（*HR*＝12.0, 95％*CI*: 2.9–50.7, *P*＝0.001）. Logistic regression analysis showed that having a CMV-negative donor status without letermovir prophylaxis was an independent risk factor for refractory CMV viremia（*P*＝0.005）.

**Conclusion:**

CMV-negative donors increase the risk of CMV-related events in recipients. Although letermovir prophylaxis mitigates this risk, the incidence of refractory CMV viremia remains higher in the CMV-negative donor group than in the collateral CMV-positive donor group, whereas the overall survival outcomes are comparable between the two donor sources.

目前，异基因造血干细胞移植（allogeneic hematopoietic stem cell transplantation，allo-HSCT）是恶性血液肿瘤唯一可能治愈的方式。巨细胞病毒（cytomegalovirus，CMV）感染是allo-HSCT的常见并发症，可导致肺炎、肠炎、脑炎、眼炎等严重疾病，甚至增加移植相关死亡率[1]。移植后CMV血症的发生率为55％～70％[2]–[3]，CMV疾病发生率为6.3％～7.9％[3]–[4]。移植前供受者CMV血清学状况对受者CMV再激活风险有显著影响。既往流行病学研究显示，中国人群中CMV血清阳性率高达90％以上[1]。有研究报道，CMV血清阳性患者接受CMV阴性供者来源的移植物可能导致CMV反复激活[2],[5]、移植后CMV特异性免疫重建延迟[2],[5]–[7]、CMV疾病发生率升高等[4]–[5]。因此，在选择供者时，CMV血清学匹配是降低移植后CMV感染的重要策略。

当与CMV血清阳性受者血清学匹配的供者不可得时，旁系血清学匹配的供者可能成为替代选择。有报道显示，旁系供者移植物抗宿主病（GVHD）发生率高于直系供者，抗胸腺细胞球蛋白（ATG）联合微小剂量环磷酰胺方案已显著改善其疗效与安全性[8]–[11]。旁系供者的CMV血清学状态同样影响移植结局：若受者为CMV阳性，选择CMV阳性的旁系供者可能通过“免疫记忆”效应降低CMV再激活风险；若受者为CMV阴性，则需优先考虑CMV阴性旁系供者以避免原发感染。这一矛盾凸显了供者选择中需综合评估CMV血清学、HLA匹配及免疫重建能力的复杂性，也对个体化移植策略的制定提出了挑战。2022年来特莫韦在国内上市后已明显改变了CMV预防模式（2017年至2021年为来特莫韦前时代，2022年至2025年初为来特莫韦时代），CMV血症发生率显著下降，来特莫韦是否改变了供者选择策略需要更多数据支持。本研究收集了2017年至2025年于我院接受allo-HSCT的CMV阴性供者（均为单倍体移植）和旁系供者（均为单倍体移植、供者CMV血清学阳性、除外二次移植）的临床资料，分析两种供者对移植预后的影响，为临床决策提供证据。

## 病例与方法

1. 病例：本研究患者纳入标准：①2017年1月至2025年3月于北京大学人民医院血液科接受CMV血清学阴性供者（CMV抗体IgG和IgM阴性）和旁系CMV阳性非一级亲属供者来源移植物allo-HSCT的恶性血液肿瘤或重型再生障碍性贫血患者（除外二次移植）。②患者CMV血清学均为阳性，移植类型为单倍体移植。该研究已获得北京大学人民医院医学研究伦理委员会批准（批件号：2021PHB423-001）。

2. 预处理方案：①单倍体、年轻和一般状态良好的急性白血病和骨髓增生异常综合征（MDS）患者采用改良白消安（Bu）/环磷酰胺（Cy）+ ATG或全身照射（TBI）+ Cy + ATG方案。②单倍体、年老、造血干细胞移植合并症指数（HCT-CI）评分3分以上或医师认为各脏器评估无法耐受Bu/Cy+ATG或TBI+Cy+ATG方案的患者采用Bu/Cy/氟达拉滨（Flu）+ATG方案[12]–[15]。

3. GVHD的预防和治疗：GVHD的预防和治疗参照既往研究[12]–[15]。

4. CMV感染的相关定义、预防、监测、诊断及治疗：患者外周血浆CMV DNA>1 000拷贝数/ml定义为CMV血症。患者外周血浆CMV DNA<1 000拷贝数/ml维持至少1周定义为外周血浆CMV DNA转阴。难治性CMV血症定义为一线足量抗病毒治疗2周，外周血浆CMV未转阴。CMV疾病指在各器官的活检组织及体液中检查出CMV，同时表现出相应器官受累的临床症状。移植前供受者均接受CMV抗体检测。所有患者从预处理开始至回输前2 d接受更昔洛韦静脉滴注（10 mg·kg^−1^·d^−1^）预防CMV感染，移植后予阿昔洛韦预防性抗病毒治疗。2022年以后单倍体移植患者接受来特莫韦预防CMV感染至移植后100 d左右，来特莫韦与环孢素合用时剂量为240 mg/d，未合用时剂量为480 mg/d。患者移植后半年内每周检测1～2次外周血浆CMV DNA，接受单倍体移植的患者外周血浆CMV DNA阳性即开始一线抗CMV治疗，包括更昔洛韦10 mg·kg^−1^·d^−1^或膦甲酸钠90～120 mg·kg^−1^·d^−1^。一线抗CMV治疗后10～14 d外周血浆检测CMV DNA未下降，则考虑联合抗病毒治疗，包括更昔洛韦、膦甲酸钠、CMV丙种球蛋白、CMV特异性细胞毒性T淋巴细胞（CMV-CTL）治疗等，CMV-CTL的制备流程参照文献[16]。

5. 定义：移植后患者连续3 d外周血ANC>0.5×10^9^/L定义为粒细胞植入，ANC>0.5×10^9^/L的第1天定义为粒细胞植活时间。患者在脱离血小板输注的情况下，连续7 d PLT>20×10^9^/L为血小板植入，第1天定义为血小板植活时间。总生存（OS）期定义为自受者接受供者造血干细胞回输至死亡或随访截止的时间。复发时间定义为自受者接受造血干细胞回输至血液学复发的时间。非复发相关死亡（NRM）时间定义为自受者接受造血干细胞回输至非复发死亡的时间。一级亲属指与患者共享1/2基因的亲属，指父母、子女、兄弟姐妹；二级亲属指共享1/4基因的亲属，包括叔伯/姑姨、侄子/侄女、同父异母或同母异父的兄弟姐妹等；三级亲属指与患者共享1/8基因的亲属，包括表/堂兄弟姐妹等。旁系非一级亲属指旁系二级或三级亲属。

6. 随访：随访截止时间为2026年1月9日，采取查阅住院、门诊病历和（或）电话随访方式。观察指标为患者生存情况及疾病缓解状态。总体中位随访时间为1 091（660，1 536）d。

7. 统计学处理：定性资料用例数（百分比）表示，定量资料用中位数*M*（*Q*_1_，*Q*_3_）表示。连续变量资料检验是否符合正态分布，符合正态分布的资料采用独立样本*t*检验，不符合正态分布或方差齐性的数据使用非参数检验。分类变量采用卡方检验或Fisher检验。患者CMV血症出现时间、持续时间、OS的分析采用Kaplan-Meier法，采用R软件的竞争风险模型分析NRM和累积复发率（CIR）。中位随访时间利用Reverse Kaplan-Meier法，失访编码为1，结局事件编码为0，采用Kaplan-Meier法计算随访时间。

以事件的发生时间和事件发生作为研究对象建立Cox回归模型，将年龄、性别、疾病类型、危险度分层、疾病状态、供者类型、供受者关系、预处理方案、供受者血型、回输单个核细胞（MNC）和CD34^+^细胞数、血小板植活、Ⅱ～Ⅳ度急性GVHD（aGVHD）、CMV持续时间、CMV疾病纳入单因素分析，利用Cox回归模型分析OS的危险因素，利用Fine-Gray竞争风险模型将NRM作为CMV疾病的竞争事件分析CMV疾病发生的独立危险因素。将单因素分析中*P*<0.2的影响因素和供者类型进一步纳入多因素分析。纳入研究的患者中仅1例旁系CMV阳性供者粒系未植活，虽然粒系植活对患者生存至关重要，但由于例数过少难以得出可靠结论，故单因素分析和多因素分析未将其纳入。通过倾向性得分匹配（PSM）分析将年龄、移植物来源、CMV阴性供者、旁系供者进行得分匹配后进一步分析CMV相关事件。应用SPSS 22.0软件和R 4.5.1进行统计学分析。*P*<0.05为差异有统计学意义。

## 结果

1. 基线特征：本研究共纳入126例患者，所有患者均接受单倍体移植，来特莫韦前时代CMV血清学阴性供者（CMV阴性供者）组16例，供者均为一级亲属，旁系非一级亲属供者组供者CMV血清学均为阳性，与受者血清学匹配，称为旁系CMV阳性供者组，共58例；来特莫韦时代CMV阴性供者组27例，旁系CMV阳性供者组25例，四组患者的基线资料见表1。患者中位发病年龄（*P*＝0.001）和移植物来源（*P*<0.001）的差异有统计学意义，其余临床特征的差异均无统计学意义（*P*值均>0.05）（表1）。

**表1:** CMV阴性供者组和旁系CMV阳性供者组患者的基线特征比较

临床特征	来特莫韦在国内上市前		来特莫韦在国内上市后		*χ*^2^值^a^	*P*值^a^
	CMV阴性供者组（16例）	旁系CMV阳性供者组（58例）	CMV阴性供者组（27例）	旁系CMV阳性供者组（25例）		
年龄［*M*（*Q*_1_，*Q*_3_）］	38（32，43）	34（30，38）	46（36，55）	39（33，44）	16.123	0.001
男性［例（％）］	9（56.3）	27（46.6）	13（48.1）	17（68.0）	3.525	0.318
疾病类型［例（％）］					14.697	0.100
髓系肿瘤	6（37.5）	31（53.4）	18（66.7）	15（60.0）		
淋系肿瘤	10（62.5）	25（43.1）	7（25.9）	10（40.0）		
SAA	0（0）	0（0）	2（7.4）	0（0）		
混合细胞白血病	0（0）	2（3.4）	0（0）	0（0）		
移植前疾病状态［例（％）］					16.890	0.154
CR1	13（81.3）	48（82.8）	20（74.1）	17（68.0）		
CR2	1（6.3）	3（5.2）	1（3.7）	2（8.0）		
≥CR3	0（0）	5（8.6）	0（0）	0（0）		
NR	1（6.3）	1（1.7）	1（3.7）	1（4.0）		
原发病状态	1（6.3）	1（1.7）	5（18.5）	5（20.0）		
供受者性别［例（％）］					16.344	0.060
男供男	4（25.0）	22（37.9）	6（22.2）	15（60.0）		
男供女	3（18.8）	21（36.2）	11（40.7）	3（12.0）		
女供男	5（31.3）	10（17.2）	7（25.9）	3（12.0）		
女供女	4（25.0）	5（8.6）	3（11.1）	4（16.0）		
HLA相合度［例（％）］					–	–
全相合	0（0）	0（0）	0（0）	0（0）		
单倍体	16（100）	58（100）	27（100）	25（100）		
供受者ABO血型［例（％）］					6.029	0.737
全相合^b^	9（56.3）	30（51.7）	17（63.0）	11（44.0）		
主要不合^c^	3（18.8）	11（19.0）	3（11.1）	7（28.0）		
次要不合^d^	4（25.0）	13（22.4）	4（14.8）	4（16.0）		
双向不合^e^	0（0）	4（6.9）	3（11.1）	3（12.0）		
移植物来源［例（％）］					36.644	<0.001
骨髓+外周血	13（81.3）	31（53.4）	4（14.8）	1（4.0）		
外周血	3（18.8）	27（46.6）	23（85.2）	24（96.0）		
回输MNC［×10^8^/kg，*M*（*Q*_1_，*Q*_3_）］	8.2（7.9，8.6）	8.8（7.4，10.6）	8.9（8.0，10.2）	8.7（8.0，10.7）	7.543	0.066
回输CD34^+^细胞中位数［×10^6^/kg，*M*（*Q*_1_，*Q*_3_）］	2.0（1.8，2.8）	2.8（1.9，4.3）	2.5（1.9，4.5）	3.0（2.5，5.8）	6.285	0.076
随访时间［d，*M*（*Q*_1_，*Q*_3_）］	1 388（217，2 133）	900（273，1 711）	1 028（607，1 134）	660（360，954）	6.974	0.073

**注** CMV：巨细胞病毒；SAA：重型再生障碍性贫血；CR1：第1次完全缓解；CR2：第2次完全缓解；≥CR3：3次及以上完全缓解；NR：未缓解；HLA：人类白细胞抗原；MNC：单个核细胞；–：无数据。^a^四组比较的结果；^b^供者和受者血型相同，如供者和受者均为A型；^c^供者有受者不具备的血型抗原，如供者为A、B或AB型，受者为O型；^d^供者具有受者不具备的血型抗体，如供者为A、B或O型，受者为AB型；^e^供者和受者的血型抗原和抗体都不同，如供者为A型，受者为B型

2. 旁系CMV阳性和阴性供者对植入的影响：无论是否使用来特莫韦预防，CMV阴性供者组患者粒系均成功植活，中位植活时间均为移植后12 d。来特莫韦前时代的旁系CMV阳性供者组57例（98.3％）粒系植活，粒系植活的中位时间为移植后14（95％*CI*：13～14）d，其中1例患者移植后2.5个月DNA指纹图显示为完全受者型，供者移植物被排斥；来特莫韦时代旁系CMV阳性供者组患者粒系均成功植活，中位植活时间为移植后14（95％*CI*：13～15）d。来特莫韦前时代CMV阴性供者组和旁系CMV阳性供者组血小板植活的中位时间分别为移植后21（95％*CI*：13～29）d和16（95％*CI*：13～19）d（*P*＝0.408）；来特莫韦时代CMV阴性供者组和旁系CMV阳性供者组的血小板植活中位时间分别为移植后14（95％*CI*：13～15）d和19（95％*CI*：17～21）d（*P*＝0.109）。

3. GVHD：来特莫韦前时代CMV阴性供者组和旁系CMV阳性供者组患者移植后Ⅱ～Ⅳ度aGVHD的累积发生率分别为6.2（95％*CI*：0～18.4）％和15.5（95％*CI*：6.1～24.9）％（*P*＝0.341）；来特莫韦时代CMV阴性供者组和旁系CMV阳性供者组患者移植后Ⅱ～Ⅳ级aGVHD的累积发生率分别为0和12.0（95％*CI*：0～24.7）％（*P*＝0.066）。来特莫韦前时代CMV阴性供者组和旁系CMV阳性供者组慢性GVHD（cGVHD）的累积发生率分别为32.6（95％*CI*：5.5～59.7）％和34.4（95％*CI*：20.7～48.9）％（*P*＝0.984）；来特莫韦时代CMV阴性供者组和旁系CMV阳性供者组cGVHD的累积发生率分别为17.5（95％*CI*：0～33.2）％和41.3（95％*CI*：20.1～62.5）％（*P*＝0.272）。

4. CMV相关事件：来特莫韦前时代，CMV阴性供者组难治性CMV血症发生率明显高于旁系CMV阳性供者组（87.5％对50.0％，*P*＝0.007），但CMV疾病发生率（25.0％对10.3％，*P*＝0.129）的差异无统计学意义。来特莫韦前时代CMV阴性供者组（16例）发生的4例CMV疾病均为CMV肺炎，1例支气管灌洗液CMV DNA阳性，余3例均为临床拟诊，最终结局为1例死于CMV肺炎，1例死于复发；旁系CMV阳性供者组（58例）发生的6例CMV疾病中，2例CMV眼炎，1例CMV肠炎，3例CMV肺炎，其中1例死于复发。CMV阴性供者组首次CMV血症持续中位时间较旁系CMV阳性供者组长［29（95％*CI*：25～33）d对17（95％*CI*：15～19）d，*P*＝0.004］。CMV阴性供者组患者移植后CMV激活次数≥3次的比例为50.0％（8/16），明显高于旁系CMV阳性供者组（6.9％，4/58）（*P*<0.001）。

来特莫韦时代，CMV阴性供者组患者移植后首次出现CMV血症的中位时间从移植后37（95％*CI*：31～43）d推迟到了移植后140（95％*CI*：136～144）d（*P*<0.001），旁系CMV阳性供者组则从移植后36（95％*CI*：33～39）d推迟到了移植后111（95％*CI*：76～146）d（*P*＝0.013）。来特莫韦时代，CMV阴性供者组和旁系CMV阳性供者组首次CMV血症持续中位时间分别为18（95％*CI*：17～19）d和12（95％*CI*：0～25）d，差异无统计学意义（*P*＝0.313）。来特莫韦的应用虽然降低了CMV阴性供者组难治性CMV血症的发生率（40.7％对87.5％，*P*＝0.003），但依然高于旁系CMV阳性供者组（40.7％对12.0％，*P*＝0.020）。CMV阴性供者组和旁系CMV阳性供者组CMV疾病发生率（7.4％对20.0％，*P*＝0.184）和移植后CMV激活次数≥3的比例（7.4％对8.0％，*P*＝0.936）的差异均无统计学意义。

5. PSM分析：来特莫韦时代，CMV阴性供者组患者移植后首次出现CMV血症的中位时间从移植后43（95％*CI*：33～53）d推迟到了移植后131（95％*CI*：121～141）d（*P*＝0.009），旁系CMV阳性供者组则从移植后35（95％*CI*：29～41）d推迟到了移植后111（95％*CI*：76～146）d（*P*<0.001）。来特莫韦时代，CMV阴性供者组和旁系CMV阳性供者组首次CMV血症持续中位时间分别为18（95％*CI*：0～36）d和12（95％*CI*：0～25）d，两者的差异无统计学意义（*P*＝0.859）。来特莫韦前时代，CMV阴性供者组和旁系CMV阳性供者组难治性CMV血症的发生率分别为71.4％和45.8％（*P*＝0.233）；来特莫韦时代两组难治性CMV血症发生率分别为42.9％和12.5％（*P*＝0.074）。

6. 生存结局：中位随访1 091（660，1 536）d，来特莫韦前时代，CMV阴性供者组和旁系CMV阳性供者组3年OS率［75.0（95％*CI*：53.8～96.2）％对80.0（95％*CI*：68.6～91.4）％，*P*＝0.479］、3年累积NRM率［19.6（95％*CI*：0～39.6）％对9.8（95％*CI*：1.8～17.8）％，*P*＝0.278］和3年CIR［6.7（95％*CI*：0～19.2）％对19.8（95％*CI*：8.0～31.0）％，*P*＝0.291］的差异均无统计学意义。来特莫韦时代，CMV阴性供者组和旁系CMV阳性供者组的3年OS率［88.3（95％*CI*：75.8～100.0）％对76.4（95％*CI*：58.0～94.8）％，*P*＝0.622］、3年累积NRM率［11.7（95％*CI*：0～24.2）％对20.1（95％*CI*：2.8～37.4）％，*P*＝0.865］和3年CIR［11.8（95％*CI*：0～24.3）％对8.2（95％*CI*：0～19.0）％，*P*＝0.629］的差异也均无统计学意义。

7. 多因素分析：多因素分析显示，供者类型不影响患者移植后OS和NRM，但CMV疾病是影响患者OS（*HR*＝3.0，95％*CI*：1.3～7.3，*P*＝0.014）和NRM（*HR*＝3.1，95％*CI*：1.1～8.8，*P*＝0.035）的独立危险因素（表2、3）。将NRM作为CMV疾病的竞争事件，CMV疾病发生的独立危险因素为难治性CMV血症（*HR*＝12.0，95％*CI*：2.9～50.7，*P*＝0.001）（表4）。Logistic回归分析显示，难治性CMV血症发生的独立危险因素为无来特莫韦预防的CMV阴性供者（*P*＝0.005）（表5）。

**表2:** 影响患者接受异基因造血干细胞移植后总生存的单因素和多因素分析

因素	单因素分析		多因素分析	
	*HR*（95％*CI*）	*P*值	*HR*（95％*CI*）	*P*值
年龄	1.0（1.0～1.1）	0.022	–	–
性别（女，男）	0.5（0.2～1.2）	0.118	–	–
疾病类型	–	0.290	–	–
淋系肿瘤，髓系肿瘤	1.3（0.6～3.1）	0.549	–	–
SAA，髓系肿瘤	5.0（0.6～39.1）	0.122	–	–
疾病状态（≥CR3+NR，CR1+CR2+MDS）	5.8（1.9～17.4）	0.002	–	–
供者类型	–	0.754	–	–
无LET旁系供者，无LET CMV阴性供者	0.7（0.2～2.2）	0.534	–	–
LET CMV阴性供者，无LET CMV阴性供者	0.4（0.1～2.0）	0.283	–	–
LET旁系供者，无LET CMV阴性供者	0.8（0.2～2.9）	0.708	–	–
预处理方案（标准剂量，减低剂量）	0.3（0.1～0.8）	0.016	0.3（0.1～0.7）	0.008
供受者性别	–	0.499	–	–
男供女，男供男	0.8（0.3～2.3）	0.717	–	–
女供男，男供男	1.4（0.5～3.8）	0.556	–	–
女供女，男供男	0.3（0～2.2）	0.231	–	–
供受者ABO血型	–	0.488	–	–
主要不合，全相合	1.5（0.6～4.1）	0.408	–	–
次要不合，全相合	0.5（0.1～2.2）	0.354	–	–
双向不合，全相合	1.6（0.4～5.7）	0.478	–	–
移植物来源（骨髓+外周血，外周血）	1.3（0.6～3.0）	0.565	–	–
回输MNC数	0.9（0.7～1.1）	0.237	–	–
回输CD34^+^细胞数	1.0（0.9～1.1）	0.801	–	–
血小板植活（是，否）	0.1（0～0.3）	<0.001	0.1（0～0.2）	<0.001
Ⅱ～Ⅳ级aGVHD（是，否）	1.3（0.4～4.5）	0.649	–	–
CMV疾病（是，否）	4.4（1.8～10.6）	0.001	3.0（1.3～7.3）	0.014

**注** SAA：重型再生障碍性贫血；≥CR3：3次及以上完全缓解；NR：未缓解；CR1：第1次完全缓解；CR2：第2次完全缓解；MDS：骨髓增生异常综合征；LET：来特莫韦预防；CMV：巨细胞病毒预防；MNC：单个核细胞；aGVHD：急性移植物抗宿主病；–：无数据

**表3:** 患者接受异基因造血干细胞移植后非复发相关死亡的单因素和多因素分析

因素	单因素分析		多因素分析	
	*HR*（95％*CI*）	*P*值	*HR*（95％*CI*）	*P*值
年龄	1.1（1.0～1.1）	0.002	–	–
年龄≥42岁，年龄<42岁	3.7（1.3～10.1）	0.012	–	–
性别（女，男）	0.5（0.2～1.6）	0.254	–	–
疾病类型	–	0.124	–	–
淋系肿瘤，髓系肿瘤	0.6（0.2～1.8）	0.342	–	–
SAA，髓系肿瘤	5.6（0.7～43.6）	0.103	–	–
疾病状态（≥CR3+NR，CR1+CR2+MDS）	3.7（0.8～16.8）	0.087	–	–
供者类型	–	0.714	–	–
无LET旁系供者，无LET CMV阴性供者	0.5（0.1～1.9）	0.289	–	–
LET CMV阴性供者，无LET CMV阴性供者	0.6（0.1～2.9）	0.519	–	–
LET旁系供者，无LET CMV阴性供者	0.8（0.2～3.6）	0.780	–	–
预处理方案（标准剂量，减低剂量）	0.2（0.1～0.5）	0.001	0.1（0～0.4）	0.001
供受者性别	–	0.721	–	–
男供女，男供男	1.3（0.4～4.4）	0.701	–	–
女供男，男供男	2.1（0.6～7.1）	0.257	–	–
女供女，男供男	0	0.979	–	–
供受者ABO血型（主要不合+双向不合，其他）	2.3（0.8～6.3）	0.114	–	–
移植物来源（骨髓+外周血，外周血）	1.4（0.5～3.7）	0.562	–	–
回输MNC数	0.9（0.7～1.2）	0.432	–	–
回输CD34^+^细胞数	1.0（0.9～1.1）	0.786	–	–
血小板植活（是，否）	0.1（0～0.4）	<0.001	0.1（0～0.3）	<0.001
Ⅱ～Ⅳ度aGVHD（是，否）	2.1（0.6～7.3）	0.259	–	–
CMV疾病（是，否）	4.9（1.7～13.7）	0.003	3.1（1.1～8.8）	0.035

**注** SAA：重型再生障碍性贫血；≥CR3：3次及以上完全缓解；NR：未缓解；CR1：第1次完全缓解；CR2：第2次完全缓解；MDS：骨髓增生异常综合征；LET：来特莫韦预防；CMV：巨细胞病毒；MNC：单个核细胞；aGVHD：急性移植物抗宿主病；–：无数据

**表4:** 患者接受异基因造血干细胞移植后发生CMV疾病的危险因素分析

因素	单因素分析		多因素分析	
	*OR*（95％ *CI*）	*P*值	*OR*（95％ *CI*）	*P*值
年龄	1.0（1.0～1.1）	0.044	1.0（1.0～1.1）	0.042
性别（女，男）	1.1（0.4～3.0）	0.783	–	–
疾病类型	–	0.998	–	–
淋系肿瘤，髓系肿瘤	1.0（0.4～2.6）	0.952	–	–
SAA，髓系肿瘤	0（0）	0.985	–	–
疾病状态（≥CR3+NR，CR1+CR2+MDS）	3.3（0.7～14.6）	0.123	–	–
供者类型	–	0.336	–	0.061
无LET旁系供者，无LET CMV阴性供者	0.4（0.1～1.4）	0.154	0.8（0.2～2.8）	0.694
LET CMV阴性供者，无LET CMV阴性供者	0.3（0.1～1.5）	0.135	0.3（0～1.6）	0.154
LET旁系供者，无LET CMV阴性供者	0.7（0.2～2.7）	0.624	2.8（0.6～14.1）	0.199
预处理方案（标准剂量，减低剂量）	0.4（0.1～1.0）	0.052	–	–
供受者性别	–	0.583	–	–
男供女，男供男	2.2（0.6～7.5）	0.206	–	–
女供男，男供男	2.1（0.5～8.5）	0.285	–	–
女供女，男供男	1.3（0.2～7.1）	0.760	–	–
供受者ABO血型（主要不合+双向不合，其他）	1.1（0.4～3.1）	0.869	–	–
移植物来源（骨髓+外周血，外周血）	1.5（0.6～3.8）	0.428	–	–
回输MNC数	0.9（0.7～1.2）	0.562	–	–
回输CD34^+^细胞数	1.1（1.0～1.3）	0.189	–	–
血小板植活（是，否）	0.5（0.1～2.1）	0.341	–	–
CMV感染前Ⅱ～Ⅳ度aGVHD（是，否）	1.4（0.2～10.3）	0.761	–	–
CMV血症持续时间≥35 d（是，否）	11.8（4.1～33.7）	<0.001	–	–
难治性CMV血症（是，否）	6.4（1.8～22.3）	0.004	12.0（2.9～50.7）	0.001

**注** CMV：巨细胞病毒；SAA：重型再生障碍性贫血；≥CR3：3次及以上完全缓解；NR：未缓解；CR1：第1次完全缓解；CR2：第2次完全缓解；MDS：骨髓增生异常综合征；LET：来特莫韦预防；MNC：单个核细胞；aGVHD：急性移植物抗宿主病；–：无数据

**表5:** 影响患者接受异基因造血干细胞移植后发生难治性CMV血症的危险因素分析

因素	单因素分析		多因素分析	
	*OR*（95％ *CI*）	*P*值	*OR*（95％ *CI*）	*P值*
年龄	1.0（0.9～1.0）	0.806	–	–
性别（女，男）	2.1（1.0～4.4）	0.037	–	–
疾病类型	–	0.515	–	–
淋系肿瘤，髓系肿瘤	1.5（0.7～3.1）	0.249	–	–
SAA，髓系肿瘤	0（0）	0.999	–	–
疾病状态（≥CR3+NR，CR1+CR2+MDS）	1.2（0.3～5.1）	0.780	–	–
供者类型	–	0.001	–	0.005
无LET旁系供者，无LET CMV阴性供者	0.1（0～0.7）	0.015	0.1（0～0.6）	0.011
LET CMV阴性供者，无LET CMV阴性供者	0.1（0～0.5）	0.006	0.1（0～0.7）	0.020
LET旁系供者，无LET CMV阴性供者	0（0～0.1）	<0.001	0（0～0.2）	<0.001
预处理方案（标准剂量，减低剂量）	0.8（0.3～2.1）	0.641	–	–
供受者性别	–	0.152	–	0.054
男供女，男供男	1.7（0.7～3.9）	0.245	1.2（0.5～3.2）	0.713
女供男，男供男	0.5（0.2～1.5）	0.228	0.2（0～0.8）	0.018
女供女，男供男	1.7（0.6～5.5）	0.345	1.1（0.3～4.6）	0.902
供受者ABO血型（主要不合+双向不合，其他）	0.7（0.3～1.5）	0.338	–	–
移植物来源（骨髓+外周血，外周血）	3.9（1.8～8.4）	<0.001	2.3（0.9～6.0）	0.088
回输MNC数	0.8（0.7～1.0）	0.045	–	–
回输CD34^+^细胞数	1.0（1.0～1.2）	0.511	–	–
血小板植活（是，否）	1.7（0.5～6.1）	0.388	–	–
CMV感染前Ⅱ～Ⅳ度aGVHD（是，否）	1.2（0.2～6.3）	0.810	–	–

**注** CMV：巨细胞病毒；SAA：重型再生障碍性贫血；≥CR3：3次及以上完全缓解；NR：未缓解；CR1：第1次完全缓解；CR2：第2次完全缓解；MDS：骨髓增生异常综合征；LET：来特莫韦预防；MNC：单个核细胞；aGVHD：急性移植物抗宿主病；–：无数据

## 讨论

本回顾性研究收集了2017年至2021年（来特莫韦前时代）和2022年至2025年初（来特莫韦时代）我院接受allo-HSCT的CMV阴性供者组和旁系CMV阳性供者组的临床资料。来特莫韦的应用显著推迟了CMV阴性供者组和旁系CMV阳性供者组患者移植后首次CMV血症的发生时间，并消除了两组患者首次CMV血症持续中位时间的差异。来特莫韦的应用显著降低了CMV阴性供者组难治性CMV血症的发生率（40.7％对87.5％，*P*＝0.003），但仍高于旁系CMV阳性供者组（40.7％对12.0％，*P*＝0.020）。两组的GVHD发生率、3年OS率和NRM率的差异均无统计学意义。

本研究发现，来特莫韦前时代供受者CMV血清学状态不匹配会导致CMV相关事件增多，包括难治CMV血症发生率升高，CMV感染时间延长，但并未对移植最终结局（OS率、NRM率和CIR）产生影响，与欧洲血液与骨髓移植学会（EBMT）发表的结果一致。在单倍体移植和同胞全相合移植模式下，CMV血清学阳性受者选取血清学阴性供者对预后无影响[17]–[18]，但也有相反报道[10],[19]–[20]。原因可能为各移植中心预处理方案不同，对免疫重建的影响有差异[10],[19]–[20]，且各中心对CMV血症治疗手段不一，近年来CMV-CTL的应用也提高了CMV疾病的治疗水平，也可能是CMV相关事件虽然增多，但并未影响移植结局的原因之一。

多项基础研究证实，CMV血清学阳性供者体内存在CMV特异性记忆T细胞，不仅可通过旁观者激活效应在移植后72 h内启动抗病毒免疫应答[21]，而且使T细胞遇到CMV抗原时能快速启动细胞因子的转录[22]，迁移到感染部位并发挥效应功能。供者免疫记忆可通过直接输注效应、交叉呈递激活、克隆多样性差异对受者T细胞重建产生重要影响[23]–[24]。因此在机制上，CMV血清学阴性供者会延迟CMV特异性免疫重建，供者来源的CMV-CTL成功制备回输给受者后，其体内存活时间可达6～12个月，且持续保持CMV特异性杀伤活性，显著降低了CMV复发率。

因CMV发生风险可能影响患者远期预后、延长住院时间、增加医疗费用[25]，仍有一定的供者选择意义，未来需要前瞻性多中心数据验证。来特莫韦时代改变了CMV的预防模式，现我中心全相合移植患者不使用来特莫韦，单倍体移植患者应用来特莫韦预防CMV感染至移植后100 d左右，但研究显示停用来特莫韦预防后，20％患者会有迟发型CMV感染，所以有移植中心将来特莫韦预防时间延迟至移植后200 d[26]，有报道显示，移植后100～200 d的CMV感染率可从19％降至3％[27]。近期研究报道，移植后100 d检测CMV特异性T细胞重建情况可区分患者是否产生保护性T细胞应答，缺乏保护性T细胞应答患者存在发生CMV疾病的风险，需延长预防治疗疗程[28]。整体而言，CMV感染高风险患者可考虑延长来特莫韦预防CMV时间至移植后200 d。

本研究的不足之处包括：本研究是单中心回顾性研究，CMV阴性供者和旁系CMV阳性供者病例数有限，可能存在选择偏倚，需要进一步增加样本量以提升分析的可信度并在临床中验证结论的可靠性；缺乏患者移植后T细胞免疫重建数据，未能从原理上深入探讨CMV血清学不匹配对患者免疫重建的影响及可能导致CMV感染时间延长、频繁再激活的机制，未来研究将补充免疫重建方向数据以进一步探讨机制。

总体来说，CMV阴性供者会增加受者发生CMV相关事件的风险。来特莫韦的应用虽可改善这一情况，但CMV阴性供者组的难治性CMV血症发生率仍高于旁系CMV阳性供者组，两种供者来源的受者总体生存结局相当。
